# Projecting Podocarpaceae response to climate change: we are not out of the woods yet

**DOI:** 10.1093/aobpla/plad034

**Published:** 2023-06-08

**Authors:** Thando C Twala, Jolene T Fisher, Kelsey L Glennon

**Affiliations:** School of Animal, Plant and Environmental Sciences, University of the Witwatersrand, Private Bag 3, Johannesburg, WITS 2050, South Africa; School of Animal, Plant and Environmental Sciences, University of the Witwatersrand, Private Bag 3, Johannesburg, WITS 2050, South Africa; School of Animal, Plant and Environmental Sciences, University of the Witwatersrand, Private Bag 3, Johannesburg, WITS 2050, South Africa

**Keywords:** Climate change, environmental niche, persistence, podocarp, species distribution

## Abstract

Under the changing climate, the persistence of Afrotemperate taxa may be threatened as suitable habitat availability decreases. The unique disjunct ranges of podocarps in southern Africa raise questions about the persistence of these species under climate change. Here, we identified likely environmental drivers of these distributions, characterized the current and future (2070) environmental niches, and projected distributions of four podocarp species in South Africa. Species distribution models were conducted using species locality data for *Afrocarpus falcatus*, *Podocarpus latifolius*, *Pseudotropheus elongatus* and *Podocarpus henkelii* and both historical climate data (1970–2000) and future climate scenarios (Representative Concentration Pathway [RCP] 4.5 and 8.5, 2061–2080) to estimate the current and future distributions. We also used this opportunity to identify the most important climatic variables that likely govern each species’ distribution. Using niche overlap estimates, a similarity test, and indices of niche expansion, stability and unfilling, we explored how niches change under different climate scenarios. The distribution of the study species was governed by the maximum temperature of the warmest month, temperature annual range, mean temperature of the wettest quarter, and precipitation of the wettest, driest and warmest quarters. The current distribution of *A. falcatus* was predicted to expand to higher elevations under RCP 4.5 and RCP 8.5. *Podocarpus henkelii* was predicted to lose most of its suitable habitat under RCP 4.5 and expand under RCP 8.5; however, this was the opposite for *P. elongatus* and *P. latifolius*. Interestingly, *P. elongatus,* which had the smallest geographic distribution, showed the most vulnerability to climate change in comparison to the other podocarps. Mapping the distribution of podocarps and understanding the differences in their current and future climate niches provide insight into potential climate drivers of podocarp persistence and the potential for adaptation of these species. Overall, these results suggest that *P. elongatus* and *P. henkelii* may expand to novel environmental niches.

## Teaser

South African podocarps' distribution and response to climate change are primarily influenced by seasonal drought and physiological constraints. Total rainfall during the summer and winter months was identified as a crucial factor influencing podocarp distribution in South Africa. Podocarps are predicted to maintain their environmental niche under climate change. Furthermore, temperature annual range and mean temperature during the wettest quarter were found to limit the future distribution of some podocarps. The study highlights the importance of understanding environmental variables that shape and maintain species' geographic distributions for conservation purposes. It provides valuable insights into conservation actions and the re-introduction of South African podocarps in suitable habitats. The findings also underscore the need for physiologically oriented distribution models to consider the role of physiological thresholds in determining podocarp distribution.

## Introduction

Conifers are a crucial component of global forests with both economic and ecological importance (Farjon 2018). Podocarpaceae (podocarp) is the second most species-rich conifer family and the largest clade in southern conifers with 19 genera and 187 species (Christenhusz and Byng 2016), and are one of the few gymnosperms that inhabit tropical forests in the Southern Hemisphere. Species in the Podocarpaceae were historically centred in Gondwana, subsequently expanding to Australasia, southernmost Africa, and currently also occur in Malaysia (Leslie *et al.* 2012; Quiroga *et al.* 2016). Podocarps were prominent in Gondwana during the Cretaceous period (Krassilov 1974) diversifying by the early Triassic period into the podocarps we know today (White 1981; Mill 1999). This diversification was possibly due to the onset of warmer and wetter climates because of the opening of the South Ocean (Atlantic and Indian Ocean; Morley 2011). During the mid-Cretaceous, podocarps went extinct in West Africa during the flattening of the uplands, reduced in geographic extent in India during the late Cretaceous when India drifted northwards (Morley 2000), and subsequently dispersed into its current Southeast Asian distribution following global cooling. In Africa, global cooling may have enabled the dispersal of podocarps from East Africa to the highlands of West Africa through northern and southern pathways (Migliore *et al.* 2022). Presently, podocarps have a largely pantropical distribution. Some taxa extend into subtropical and temperate latitudes, where they mainly occur in Australasia, Central and South America, and tropical montane Africa (Farjon 2010; Klaus and Matzke 2020). In Afrotemperate forests, podocarps persist within fynbos, heathland and grassland matrices, where fire and grass competition are the predominant factors that influence forest distribution (Adie and Lawes 2009a, 2011; Adie *et al.* 2017).

Climate change and angiosperm competition have been some of the most important factors influencing podocarp distribution and persistence (Quiroga and Premoli 2007; Migliore *et al.* 2022). Podocarps appeared to respond to these threats through persistence in refugia (Quiroga *et al.* 2018; Tesfamariam *et al.* 2022). As a result, podocarps show remarkable adaptability in habitat, growth form and physiological traits. For instance, some members of the family are semiaquatic (*Retrophyllum minor*), parasitic (*Parasitaxus usta*), exhibit resprouting and fire and drought tolerance (*Podocarpus drouynianus* and *Podocarpus spinulosus*), shade tolerance (*Podocarpus latifolius*), tolerance to soil anoxia (*Manoao colensoi*), diverse fleshy seed cones (*Podocarpus elongatus*, *Afrocarpus falcatus*), diverse leaf morphology (*Podocarpus nagi*, *P. henkelii*, *P. macrophyllus*, leaf and individual longevity, and have conspicuous root nodules which house arbuscular mycorrhiza fungi (Molloy 1995; Hill and Brodribb 1999; Biffin *et al.* 2012; Contreras *et al.* 2017). Remarkably, most podocarps are restricted to humid, mountainous environments, including angiosperm-dominated forests (Kitayama *et al.* 2011). Many extant podocarps are slow growing, fire and drought intolerant, and these characteristics put them at a competitive disadvantage when co-occurring with angiosperms (Bond 1989; Brodribb and Hill 1998), which restricts them to these ‘steppingstone’ refugia.

Developing management strategies and practical measures to conserve podocarps is difficult without identifying key environmental variables to each species’ climatic niche and predicted geographical distribution, particularly under future climate conditions. Moreover, climate change affects habitat requirements for many species (Bakkenes *et al.* 2002; Aitken *et al.* 2008; Essl *et al.* 2011; Moran 2020; Fricke *et al.* 2022). Consequently, determining whether climate change will affect the geographical distribution and environmental niches of podocarps presents another critical challenge linked to their ecological value and significance. The current distributions of *A. falcatus*, *P. elongatus, P. henkelii* and *P. latifolius* are relatively well known; however, little is known about the environmental drivers of podocarp distribution, their dispersal patterns and how climate change will affect their distribution and environmental niches.

Niche conservatism is used to test how species shift (expand and/or contract) their ranges in response to climate change (Wiens 2004; Wiens and Graham 2005; Wiens *et al.* 2010). This ecological and evolutionary process is an issue of concern particularly due to the expected adverse effects of climate change on species distributions and diversity (Araújo *et al.* 2013). Therefore, modelling the environmental niche and projecting it into geographic space allows us to test whether species exhibit environmental niche conservatism as ranges shift under climate change. Testing environmental niche conservatism is necessary to enable us to develop strategies to mitigate the negative effects of climate change and provide important ecological insights. For the purpose of this study, ‘niche conservatism’ is defined as when the predicted environmental niche tends to remain similar to the current environmental niche (Warren *et al.* 2008). Eeley *et al.* (1999) and Colyn *et al.* (2020) suggested that the ability of forest species to track suitable environmental conditions under climate change could be significantly limited by anthropogenic landscape change. Therefore, in this study, the ability of podocarps to persist under climate change will be determined by the ability of the species to track favourable niches and expand their geographic range.

Species distribution models (SDMs) are mathematical algorithms that estimate a species’ climatic niche by characterizing a species’ occurrence in relation to environmental factors (Elith *et al.* 2006). SDMs can be used to predict and analyse patterns of distribution and could estimate the risk these environmental drivers pose to species (Guisan and Thuiller 2005; Elith and Leathwick 2009; Guisan *et al.* 2013). Species distribution models quantify the relationship between plant distributions and environmental factors (Guisan and Thuiller 2005; Elith and Leathwick 2009; Wiens *et al.* 2010; Guisan *et al.* 2013); thus, demonstrating their vulnerability more accurately. Species distribution models often assume niche conservatism, which suggests that species can maintain their environmental niches (Peterson *et al.* 1999; Wiens 2004; Wiens *et al.* 2009; Peterson 2011). It is still controversial whether species’ environmental niches are preserved in space and time (Peterson *et al.* 1999; Broennimann *et al.* 2007). As a result of the ongoing theoretical development and quantification of a species’ environmental niche, researchers have advanced our understanding of how species fluctuate in their requirements for and tolerance of various factors (Soberón 2007). In this study, we used a combined environmental (niche overlap indices) and geographical approach (temporal transferability of SDMs) to characterize the distribution of *A. falcatus*, *P. elongatus*, *P. henkelii* and *P. latifolius*. The objectives of this study were to (i) identify the environmental variables that shape the distribution of *A. falcatus*, *P. elongatus*, *P. henkelii* and *P. latifolius*; (ii) model the current distribution of South African podocarps; (iii) project the current models onto two future climate emissions scenarios (Representative Concentration Pathway [RCP] 4.5 and RCP 8.5); and (iv) compare the future geographic distribution of podocarp environmental niches to their current environmental niche geographic distribution. Such comparisons will enable us to infer the vulnerability of these South African podocarps to the changing environment.

## Methods

### Study species

South Africa consists of four podocarp species from sister genera: *Afrocarpus* (*A. falcatus*) and *Podocarpus* (*P. elongatus*, *P. henkelii* and *P. latifolius*; Farjon 2001). In Africa, podocarps are restricted to highland archipelagos in montane forests along the Afromontane Forest belt, where they persist in small forest patches within a grassland, fynbos or heathland matrix. The Afromontane Forest belt extends from Ethiopia along the eastern mountain range, all the way to the southern Cape in South Africa. *Afrocarpus falcatus* is present throughout Afromontane forests and is absent in West Africa. *Afrocarpus falcatus* and *P. latifolius* are widely distributed geographically across South Africa but are restricted to Afromontane Forest habitats. In South Africa, *A. falcatus* and *P. latifolius* occur along the southwest of South Africa and extend to the south and east coasts of the country up the Drakensberg mountains in the eastern parts of the country and extend northwards in temperate midland regions towards Zimbabwe. *Podocarpus henkelii* occurs in forest habitats and is restricted to summer rainfall areas in the mesic east of the country. *Podocarpus elongatus* only occurs in the extreme western and southern parts of the country (winter rainfall) in the forest and fynbos biome.

Generally, our study included three major steps, with relevant details reported below: (i) data were downloaded, compiled and variables were selected; (ii) *A. falcatus*, *P. elongatus*, *P henkelii* and *P. latifolius* occurrence and climate data were used to characterize niche and environmental spaces; and (iii) a variety of species distribution modelling algorithms were used to project the potential distribution of *A. falcatus*, *P. elongatus*, *P henkelii* and *P. latifolius* in South Africa.

### Species occurrences

**Figure 1.**
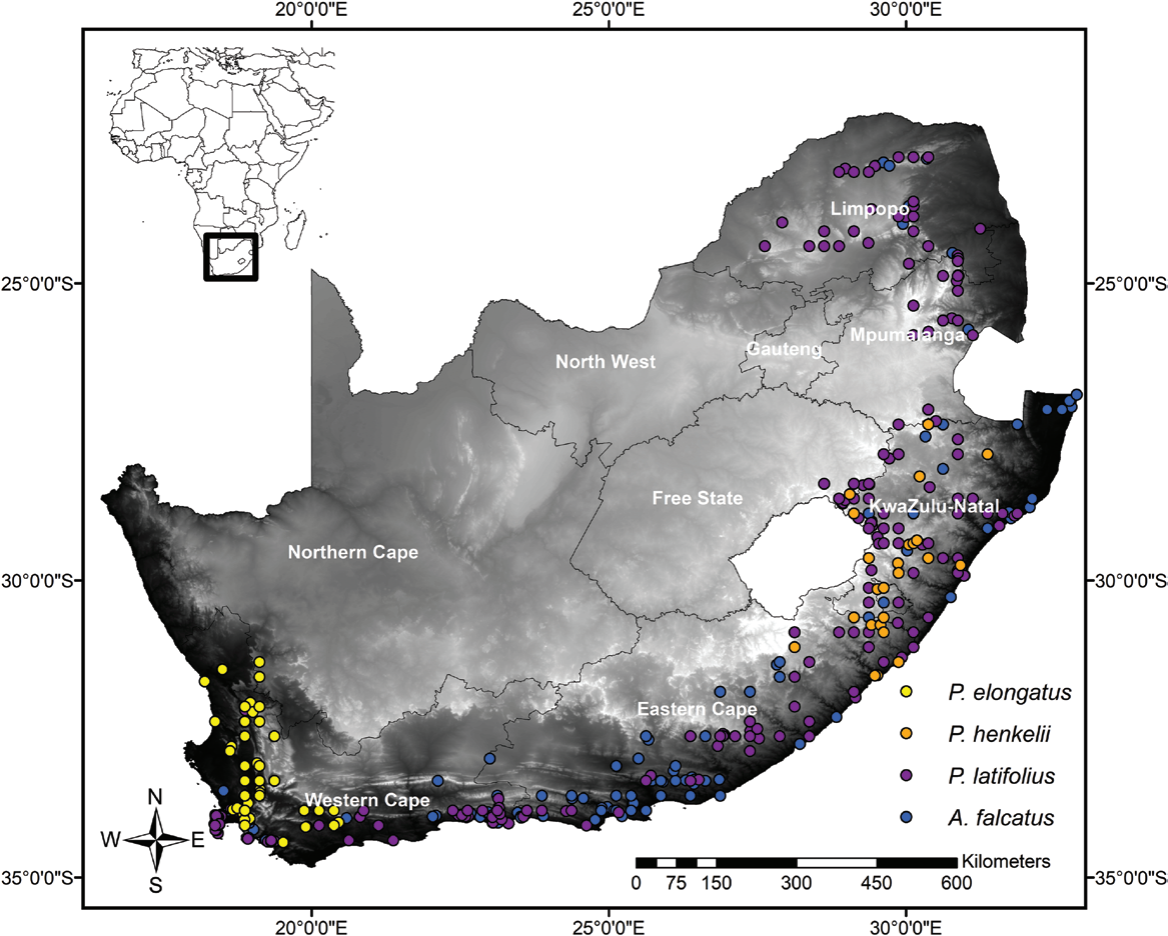
South African occurrences of *Afrocarpus falcatus*, *Podocarpus latifolius*, *P. elongatus* (inset A) and *P. henkelii* (inset B) that were downloaded from the Global Biodiversity Information Facility (http://gbif.org). The map is shaded according to elevation where darker grey indicates low elevation and light grey/white areas indicate high elevation (>1800 m.a.s.l).

*Afrocarpus falcatus*, *P. elongatus*, *P. henkelii* and *P. latifolius* occurrence data were downloaded from the Global Biodiversity Information Facility online repository (GBIF; https://www.gbif.org/). Where necessary, location information was georeferenced and duplicate localities were removed. Records were validated using Google Earth v7.1.2 and outliers associated with non-natural locations were removed. After filtering, 562 localities were retained in the final analysis for all four species: *A. falcatus* (*n* = 246), *P. latifolius* (*n* = 242), *P. elongatus* (*n* = 49) and *P. henkelii* (*n* = 25; Fig. 1).

### Climate variables

Current climate data were downloaded from Chelsa version 2.1 (historical [1970–2000] and future [2061–80) climate data were downloaded under RCPs 4.5 and 8.5 emission scenarios from the Hadley Global Environment Model 2-Atmosphere Ocean (HADGEM2-AO) circulation model which provides adequate coverage for Africa (Martin *et al.* 2011). These data were downloaded using ‘Chelsa.CMIP_5.download’ function from the *ClimDatDownloadR* R package (Karger *et al.* 2017; Jentsch *et al.* 2021). All environmental variables had a spatial resolution of 30 arc seconds (~1 km^2^ pixel area at the equator). To avoid obtaining spurious results and over-parameterization of the models due to variable multi-collinearity (Elith *et al.* 2010; Synes and Osborne 2011; Boria *et al.* 2014), variable correlations were minimized using dimension reduction techniques. A Pearson’s correlation test based on all 19 climate variables was conducted for all species’ presence points and used to exclude highly correlated variables (*r* > 0.65) from our models. To further avoid over-parameterization of the model and obtaining spurious results due to variable multi-collinearity, we used the variance inflation factor on the remaining variables to remove significantly correlated variables. Seven predictor variables were used in subsequent processing: Max Temperature of the Warmest Month (BIO5), Temperature Annual Range (BIO7), Mean Temperature of Wettest Quarter (BIO8), Mean Temperature of Coldest Quarter (BIO11), Precipitation of Wettest Quarter (BIO16), Precipitation of Driest Quarter (BIO17) and Precipitation of Warmest Quarter (BIO18). These seven predictor variables were used to model the species distribution for all the species to allow for comparisons between species and are variables used by other studies predicting the species distribution of podocarps (e.g. Bernardi *et al.* 2020; Tesfamariam *et al.* 2022).

### Species distribution modelling

Species distribution modelling uses occurrence data and environmental variables to simulate suitable/habitable environmental conditions for focus species. Here, we used 10 different algorithms available within the *biomod2* package (Thuiller *et al.* 2021) in R version 4.1.1 to obtain an ensemble of predicted distributions. An ensemble approach was used because numerous studies have shown that ensemble SDMs improve the predictive performance of SDMs in comparison to using one algorithm (Grenouillet *et al.* 2011; Guo *et al.* 2015; Bernardi *et al.* 2020; Zurell *et al.* 2020). The following algorithms were used to develop the ensemble SDMs: generalized linear model, generalized additive models, generalized boosting model/boosted regression trees (GBM), surface range envelop/BIOCLIM (SRE), classification tree analysis, artificial neural network, flexible discriminant analysis, multiple adaptive regression splines, random forest and maximum entropy. The following parameters were set for the models: one run of 500 pseudo-absence points was used on one model evaluation, split into 80 % for training and 20 % for testing for each SDM, and variable importance was determined using three permutations for the full extent of South Africa. The remaining model values were set to default values. Area under the receiver-operating curve (AUC), true skills statistics (TSS) and the continuous Boyce index (CBI) were used to assess model predictive efficiency and performance (Hanley and McNeil, 1982; Allouche *et al.* 2006). The Boyce index uses presence data only to measure how different the model predictions are from a random distribution of observed presence across the prediction gradient. Boyce index values range between −1 and +1, where positive values indicate that the model predictions are consistent with the presence data, values close to zero suggest that the model is no different from a random model and negative values indicate no occurrence when there is. All statistical analyses were performed in R using scripts from Broennimann *et al.* (2012), which are now available in the *ecospat* R package (Di Cola *et al.* 2017; Broennimann *et al.* 2020). The potential distribution for each species was projected under the RCP 4.5 and RCP 8.5 future climate emission scenarios for the year 2070 (average for 2061–80). Representative Concentration Pathways are models of greenhouse emission scenarios which predict future climate under different greenhouse emissions scenarios. RCPs 4.5 and 8.5 were chosen because RCP 4.5 greenhouse gas emissions are projected to peak around 2040 and then decline, while in RCP 8.5, greenhouse gas emissions are projected to continue to increase throughout the 21st century, respectively, a ‘best-case’ and ‘worst-case’ scenario (Meinshausen *et al.* 2011). At the current rate of emissions and mitigation processes, these are the most likely scenarios. RCP 2.6 was not included because it was unrealistic, as it assumed peak emissions by 2020 and subsequent decline requiring negative emissions (Meinshausen *et al.* 2011; Van Vuuren *et al.* 2011).

To identify the change in the geographic area of *A. falcatus*, *P. latifolius*, *P. henkelii* and *P. elongatus* binary maps were generated from SDMs, and the current and future binary models were used to calculate the change in geographic area between the current and future climate emissions scenarios. Changes in geographic area were classified as ‘loss’, ‘gain’ and ‘stable’. Loss referred to the area the species originally occupied, but no longer occupies after climate change. Gain referred to occupied areas that the species did not originally occupy and then occupy after climate change. Stable referred to areas that persisted after climate change, or also referred to as climatic microrefugia (Pang *et al.* 2021). All geographic information system analyses were done in ArcGIS v 10.8 (ESRI).

### Calculating niche overlap in environmental space

Assessing climatic niche characteristics is a powerful approach to studying niche divergence and conservatism. Broennimann *et al.* (2012) presented a principal component analysis (PCA) method which places environmental variables into a two-dimensional space identified by their first and second principal components. When testing niche divergence and niche conservatism hypotheses, niche overlap can provide a relatively reliable measurement of overlap. We quantified the degree of shared environmental niche space between the current and the future climate emission scenarios of *A. falcatus*, *P. elongatus*, *P. henkelii* and *P. latifolius*. In this study, niche overlap between the current and future climate emissions scenarios for each podocarp species was computed using the Schoener’s *D* statistic (Schoener 1968; Warren *et al.* 2008). Schoener’s *D* ranges from 0 (no overlap) to 1 (complete overlap). Results were then interpreted using the classification scheme suggested by Rödder and Engler (2011) where *D* ranges indicated potential interpretations: 0.0–0.2 = no or very limited overlap, 0.2–0.4 = low overlap, 0.4–0.6 = moderate overlap, 0.6–0.8 = high overlap and 0.8–1.0 = very high overlap/identical niche. Subsequently, niche overlap was interpreted in the context of three general niche categories: niche expansion, stability/stasis and unfilling (Petitpierre *et al.* 2012; Glennon *et al.* 2014; Guisan *et al.* 2014; Di Cola *et al.* 2017). Niche stability (i.e. the proportion of the current range niche overlapping with the future range niche), niche expansion (i.e. the proportion of the future range niche not overlapping with the current range niche) and niche unfilling (i.e. the proportion of the current range niche not occupied in the future range niche) enable the ability to generate hypotheses about the potential drivers of podocarp niche dynamics between current and predicted climatic emission scenarios. The niche stability and expansion values always sum to 100 %. The niche unfilling value corresponds to the expansion value when shifting current and future ranges. Niche conservatism is defined as the tendency for species to retain their environmental niche in space and time and is represented by ‘niche stability’.

We used an ordination technique that applies kernel density smoothers to species presences in environmental space (Broennimann *et al.* 2012) to determine the environmental niche occupied by each species under current and future climate emissions scenarios. The kernel density function is applied for smoothing the density of occurrences for each of the generated grid cells, which correspond to the vector of the available environmental conditions in the study area under each climate emission scenario. This smoothing approach removes any biases to obtain a better representation of the environmental conditions suitable for each species under current and future climate emissions scenarios. This approach was implemented using a PCA which is an ordination technique that is calibrated on the entire environmental space based on the focal variables of both current and future climate emission scenarios (hereafter referred to as PCA-env). Further details can be found in Broennimann *et al.* (2012).

We also performed the niche similarity test, which assesses if the environmental niches of each species are more similar or more dissimilar than expected by chance in comparison to each other under current and future climate scenarios, accounting for differences in the surrounding areas of the localities (background space) under current and future climate emissions scenarios (Warren *et al.* 2008; Warren *et al.* 2010, 2019). The niche similarity test compares the niche overlap of one species range randomly distributed over its background, while keeping the other unchanged (e.g. Current: *A. falcatus* → *P. elongatus*), and then carries out the reciprocal comparison (e.g. Current: *P. elongatus* → *A. falcatus*). For the niche similarity test, an α value of 0.05 was considered to indicate that niches were no more similar than expected by chance.

## Results

### Model evaluation indices

We calculated the commonly used (AUC) and the less commonly used (TSS and CBI) model evaluation indices. Model performance was good for all the emission scenarios (Table 1). AUC varied from 0.990 to 0.992, and TSS varied from 0.681 to 0.978. The CBI suggested that all the model predictions were consistent with the distribution of the actual data as they all had positive CBI values. However, CBI values for *P. elongatus* under the current emission scenario were low.

**Table 1.** Area under the ROC curve (AUC) and true skills statistics (TSS) values showing species distribution model performance and continuous Boyce index (CBI) showing how well the models fit the presence data of *Afrocarpus falcatus*, *Podocarpus latifolius*, *P. elongatus* and *P. henkelii* current and future climate emissions scenarios (RCP 4.5 and 8.5) in their South African distribution.

Species	Emission scenario	AUC	TSS	CBI
*A. falcatus*	Current	0.961	0.834	0.818
	RCP 4.5	0.958	0.814	0.806
	RCP 8.5	0.955	0.795	0.943
*P. elongatus*	Current	0.992	0.978	0.250
	RCP 4.5	0.990	0.940	0.889
	RCP 8.5	0.992	0.957	0.911
*P. henkelii*	Current	0.978	0.954	0.529
	RCP 4.5	0.945	0.858	0.732
	RCP 8.5	0.967	0.909	0.754
*P. latifolius*	Current	0.942	0.738	0.854
	RCP 4.5	0.918	0.681	0.894
	RCP 8.5	0.920	0.690	0.868

### Importance of environmental variables

Precipitation of the driest quarter (BIO17) and precipitation of the wettest quarter (BIO16) were the most important variables shaping the current distribution of *A. falcatus* and *P. latifolius*, respectively (Table 2). Temperature annual range (BIO07) was the most important variable constraining the future distribution of *A. falcatus* and *P. latifolius*. The current and future distributions of *P. elongatus* were predicted to be constrained by precipitation of the warmest quarter (BIO18). The most influential variable shaping the current distribution of *P. henkelii* was predicted to be BIO18. Interestingly, the maximum temperature of the warmest month (BIO5) was the most important variable constraining the distribution of *P. henkelii* under RCP 4.5, while BIO18 was the most important predictor of *P. henkelii* under RCP 8.5.

**Table 2.** Environmental variable importance scores (mean and standard deviation is shown for the three iterations) for *Afrocarpus falcatus*, *Podocarpus elongatus*, *P. henkelii* and *P. latifolius* species distribution models under current and future climate emissions scenarios. Variable importance scores range between 0 (low importance) and 1 (high importance). The variables represented in bold were the most important variable for each taxon under current and future conditions. BIO5—Max Temperature of Warmest Month; BIO7—Temperature Annual Range; BIO8—Mean Temperature of Wettest Quarter; BIO11—Mean Temperature of Coldest Quarter; BIO16—Precipitation of Wettest Quarter; BIO17—Precipitation of Driest Quarter; BIO18—Precipitation of Warmest Quarter.

Species	Emission Scenario	BIO5	BIO7	BIO8	BIO11	BIO16	BIO17	BIO18
*A. falcatus*	Current	0.084 (0.001)	0.188 (0.002)	0.024 (0.001)	0.157 (0.002)	0.046 (0.001)	**0.455 (0.007)**	0.116 (0.001)
	RCP 4.5	0.056 (0.001)	**0.607 (0.001)**	0.029 (0.001)	0.079 (0.001)	0.037 (0.001)	0.126 (0.002)	0.066 (0.000)
	RCP 8.5	0.029 (0.000)	**0.554 (0.008)**	0.037 (0.001)	0.059 (0.000)	0.122 (0.001)	0.084 (0.003)	0.115 (0.002)
*P. elongatus*	Current	0.035 (0.001)	0.045 (0.002)	0.168 (0.006)	0.029 (0.002)	0.121 (0.003)	0.262 (0.015)	**0.340 (0.005)**
	RCP 4.5	0.051 (0.001)	0.100 (0.002)	0.168 (0.003)	0.046 (0.002)	0.178 (0.006)	0.123 (0.005)	**0.335 (0.005)**
	RCP 8.5	0.094 (0.004)	0.154 (0.005)	**0.492 (0.011)**	0.051 (0.001)	0.104 (0.004)	0.043 (0.002)	0.063 (0.003)
*P. henkelii*	Current	0.095 (0.001)	0.010 (0.001)	0.011 (0.000)	0.040 (0.002)	0.068 (0.001)	0.222 (0.017)	**0.553 (0.017)**
	RCP 4.5	**0.414 (0.008)**	0.184 (0.003	0.122 (0.001)	0.083 (0.002)	0.100 (0.002)	0.059 (0.001)	0.040 (0.001)
	RCP 8.5	0.284 (0.003)	0.080 (0.000)	0.035 (0.001)	0.045 (0.002)	0.057 (0.001)	0.056 (0.006)	**0.544 (0.003)**
*P. latifolius*	Current	0.254 (0.009)	0.197 (0.004)	0.037 (0.001)	0.054 (0.001)	**0.281 (0.002)**	0.081 (0.001)	0.097 (0.002)
	RCP 4.5	0.078 (0.002)	**0.542 (0.001)**	0.037 (0.001)	0.099 (0.001)	0.173 (0.008)	0.028 (0.000)	0.043 (0.001)
	RCP 8.5	0.052 (0.001)	**0.585 (0.007)**	0.035 (0.001)	0.079 (0.002)	0.191 (0.003)	0.020 (0.000)	0.039 (0.001)

### South African podocarp distribution under climate change

**Figure 2.**
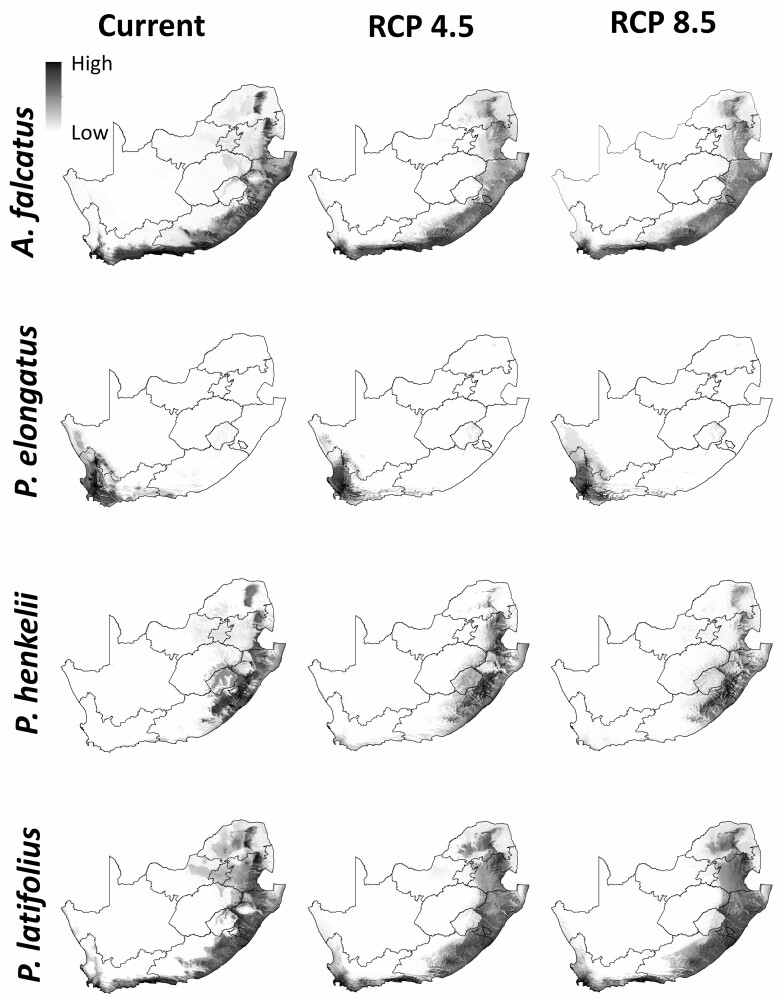
Distribution for *Afrocarpus falcatus*, *Podocarpus elongatus*, *P. henkelii,* and *P. latifolius* in South Africa predicted by using ensemble models based on climatic environmental variables under the current climate and mild (RCP 4.5) and severe (RCP 8.5) climate emission scenarios predicted for 2070. Habitat suitability ranges from high (black), moderate (grey) to low (white) suitability.

The ensemble SDM predicted high habitat suitability for *A. falcatus*, *P. elongatus*, *P. henkelii* and *P. latifolius* in the exterior part of the country, near the coastal limits and excluded the interior of the country (Fig. 2). *Podocarpus latifolius* occupied the largest current and future geographic area followed by *A. falcatus*. *Afrocarpus falcatus* and *P. latifolius* were distributed in the Limpopo, Mpumalanga, KwaZulu-Natal, Eastern Cape and Western Cape provinces, which span the summer and winter rainfall regions of the country. *Podocarpus elongatus* was restricted to the southern parts of the Western Cape, which is associated with winter rainfall. *Podocarpus henkelii* was constrained to the summer rainfall provinces of Mpumalanga, the midlands of KwaZulu-Natal and the Eastern Cape, with moderately suitable habitat in the Limpopo province. Although *P. elongatus* and *P. henkelii* had the most restricted distribution, *P. elongatus* occupied the smallest geographic area.

**Figure 3.**
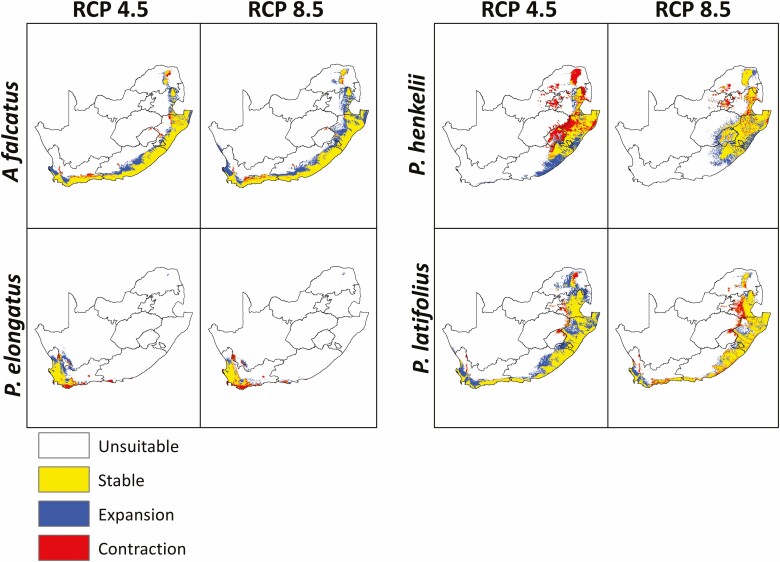
The projected impact of climate change (2070) on the habitat suitability of *Afrocarpus falcatus*, *Podocarpus latifolius*, *Podocarpus elongatus* and *Podocarpus henkelii* in South Africa under RCP 4.5 and 8.5 climate emissions scenarios.

Under climate emission scenarios, *A. falcatus* was predicted to expand to higher altitudes into the interior of the country; this was the case under RCP 4.5 and RCP 8.5 (Fig. 3). The south-western part of the distribution of *P. elongatus* was predicted to expand to higher elevations to track favourable conditions; however, the south-eastern and the northern parts of the distribution were predicted to become less suitable under both RCPs. Interestingly, the suitable habitat of *P. henkelii* under current climate was predicted to lose most of its suitable habitat under RCP 4.5 and expand its habitat to higher altitude in its northern distribution. This was the opposite for *P. latifolius*, where it gained suitable habitat under RCP 4.5 and lost suitable habitat under RCP 8.5. A common finding was that all four podocarp species expanded their habitat by moving to higher altitudes.

### Environmental niche overlap and similarity

**Figure 4.**
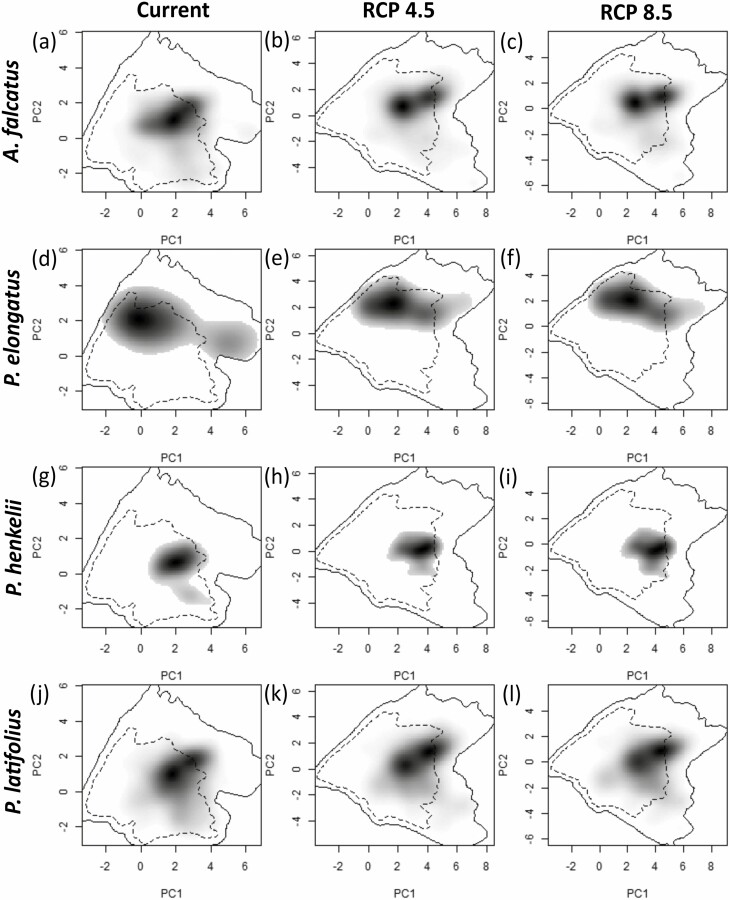
Current and future (RCP 4.5 and RCP 8.5) environmental niches of *Afrocarpus falcatus* (A–C)*, Podocarpus elongatus* (D–F)*, P. henkelii* (G–I) and *P. latifolius* (J–L). Darker shading indicates higher densities. The solid line indicates 100 % of the available environmental space and the dashed contour lines represent 50 % of the most common background space.

*Podocarpus elongatus* occurs over the largest environmental niche, while *P. henkelii* occurs over the smallest environmental niche (Fig. 4). The current environmental niches of *A. falcatus*, *P. elongatus* and *P. henkelii* are predicted to contract in 2070; however, the environmental niche of *P. latifolius* is predicted to remain stable under RCPs 4.5 and 8.5.

#### Podocarp environmental niche similarity.

The comparison of the current and future environmental niches of *A. falcatus*, *P. latifolius* and *P. henkelii* is similar to expected at random (Table 3; Fig. 4). Interestingly, the comparison of the current and RCP 4.5 environmental niche for *P. elongatus* was similar; however, the other niche comparisons between periods show niche divergence.

**Table 3.** Niche similarity *Afrocarpus falcatus*, *Podocarpus latifolius*, *P. henkelii* and *P. elongatus* under current and future climate emissions scenarios (RCP 4.5 and RCP 8.5). Significant similarity test *P*-values are in bold.

Species	Current → RCP 4.5	RCP 4.5 → Current	Current → RCP 8.5	RCP 8.5 → Current
*A. falcatus*	**0.0099**	**0.0099**	**0.0099**	**0.0198**
*P. latifolius*	**0.0198**	**0.0198**	**0.0099**	**0.0099**
*P. henkelii*	**0.0396**	**0.0198**	**0.0099**	**0.0198**
*P. elongatus*	**0.0495**	0.1089	0.1089	0.0693

#### Niche overlap between the current and future environmental niches.

The current and future environmental niches of *A. falcatus*, *P. elongatus* and *P. latifolius* showed very limited overlap (Table 4; Fig. 4). Interestingly, *P. henkelii* had the highest environmental niche overlap of the four podocarps with 59.17 % and 55.43 % overlaps between the current–RCP 4.5 and current–RCP 8.5 environmental niches, respectively.

**Table 4.** Niche overlap and dynamics between the current and future projected environmental niches of *Afrocarpus falcatus*, *Podocarpus elongatus, P. henkelii* and *P. latifolius*. Each metric ranges from zero (low) to one (high).

Species	Pairwise comparisons	Niche overlap (*D*)	Expansion	Stability	Unfilling
*A. falcatus*	Current → RCP 4.5	0.1212	0.3543	0.6456	0.1309
	Current → RCP 8.5	0.1633	0.3869	0.6131	0.1348
*P. elongatus*	Current → RCP 4.5	0.0759	0.2137	0.7863	0.4936
	Current → RCP 8.5	0.0428	0.3007	0.6993	0.6733
*P. henkelii*	Current → RCP 4.5	0.5917	0.0240	0.9760	0.3716
	Current → RCP 8.5	0.5543	0.0501	0.9499	0.4335
*P. latifolius*	Current → RCP 4.5	0.1076	0.3005	0.6995	0.0755
	Current → RCP 8.5	0.2068	0.2884	0.7116	0.0753

When the overlap between the current and future environmental niches was further broken down, we observed that the current and future environmental niches of all four podocarps were stable (Table 4). *Afrocarpus falcatus* showed greater niche expansion compared to niche unfilling. A greater extent of niche unfilling was observed for *P. elongatus* and *P. henkelii* in comparison to niche expansion for both niche comparisons (Table 4). Low niche unfilling and expansion were observed for *P. latifolius* between the current and future environmental niches.

## Discussion

This study characterized the environmental niches of South African podocarps using ordination techniques to better understand how these species may respond to the changing climate. In addition, we used ensemble SDMs to identify the environmental constraints that shape the current and future geographic distributions of *A. falcatus*, *P. elongatus*, *P. henkelii* and *P. latifolius.* We found that the ensemble SDMs accurately predicted the known geographic distributions of each podocarp species, that podocarps were generally limited by rainfall, particularly around the seed germination timeframe, and that there are likely reproductive and dispersal mechanisms as well as physiological traits that potentially prevent species from dispersing outside their current distributions.

Identifying environmental variables that shape and maintain species’ geographic distributions is an essential component of evolution and ecology. Among the seven environmental variables used for these models, total rainfall during the summer and winter months (irrespective of summer/winter rainfall region) was predicted to shape the current and future distributions of podocarps in South Africa. These findings were expected as numerous studies have identified drought stress as a factor constraining podocarp distribution and persistence (Brodribb and Hill 1998; Tesfamariam *et al.* 2022; Twala *et al.* 2022). Consequently, the distribution of podocarps in high-rainfall areas may be due to traits that reduce their ability to survive in low-rainfall regions. For instance, podocarps have small single-veined leaves that limit hydraulic conductivity (Brodribb *et al*. 2007) and recalcitrant seeds that become less viable as they lose moisture (Noel and Van Staden 1976; Dodd, Staden and Smith 1989a, 1989b; Negash 2003). Nearly all extant podocarps exhibit low to no drought tolerance (Brodribb and Hill 2004; Twala *et al.* 2022) except for the New Zealand species *P. totara*, which uses drought-avoidance strategies (Innes and Kelly 1992) and *P. macrophyllus* found across China and Japan (He *et al.* 2020). In addition, *P. latifolius* recruitment is significantly reduced under drought and deep shade where seedlings are outcompeted by grasses in the landscape (Adie and Lawes 2009b). Drought and shade are antagonistic selection pressures and together with rainfall appear to constrain podocarp distribution (Chiariello 1984; Givnish 1987; Brodribb and Hill 2000; Fiorucci and Fankhauser 2017; Lusk *et al.* 2019). Furthermore, the models indicated that South African podocarps tend to persist in moist forest patches where fires are an important driver of forest distribution, yet fires hardly occur in the patches themselves (Geldenhuys 1994; Kellman and Meave 1997).

In addition to rainfall, the future distribution of *A. falcatus* and *P. latifolius* was predicted to be limited by temperature annual range (BIO7). This finding suggests that in the future, there will be variation in seasonal temperatures where *A. falcatus* and *P. latifolius* occur across the highlands and midlands in the north-eastern parts of South Africa compared to the south-western parts of the country. In the north-eastern parts of South Africa, the models predict that the future daily temperature fluctuations will be smaller in comparison to present higher daily temperature fluctuations in the south-western parts of the country. Notably, the future distribution of *P. elongatus* was also constrained by mean temperature during the wettest quarter, which coincides with the winter months for this species as it is currently restricted to winter rainfall areas of the country. Winter rainfall and temperature are particularly important for *P. elongatus* because it produces recalcitrant seeds during the winter months and minimum rainfall and temperature requirements need to be met for recruitment and persistence. *Podocarpus henkelii* may be limited to warm high-rainfall areas because it has foliar sclereids and broad leaves (Brodribb and Hill 1998; Adie and Lawes 2011; Brodribb 2011). Furthermore, *P. henkelii* produces seeds that are more drought sensitive than *A. falcatus* seeds (Dodd *et al.* 1989a; Negash 1995).

The current and future potential distributions of podocarps obtained from our ensemble SDMs coincide with known geographic ranges (White 1981; Farjon 2001). The broad geographically distributed *P. latifolius* and *A. falcatus* co-occur with *P. elongatus* and *P. henkelii* in their respective geographic ranges; however, *P. elongatus* and *P. henkelii* do not co-occur. *Podocarpus latifolius* had the broadest geographic distribution followed by *A. falcatus*. Both *A. falcatus* and *P. latifolius* were predicted to co-occur in the north-eastern part of South Africa, along the southern coast, and through to the southwest of South Africa. This finding coincides with the actual distribution of each species. The geographic range extends across the savanna, grassland, forest, fynbos and Albany thicket biomes, and extends to the Indian Ocean coastal belt. *Afrocarpus falcatus* is the only one of these four podocarps that persists in the dry forest of the coastal lowlands (Adie and Lawes 2011; Coomes and Bellingham 2011), and this occurrence is likely as a relic from population expansion during the cool and moist conditions of the last glaciation (Finch and Hill 2008; Neumann *et al.* 2008). *Podocarpus elongatus* was predicted to occupy the smallest geographic area of all the examined podocarp species under current and future climate emissions scenarios. This taxon is presently distributed in the south and south-western parts of South Africa within the fynbos and succulent karoo biomes. *Podocarpus elongatus* can persist in this fire-prone habitat due to its ability to resprout after fire (Midgley *et al.* 1995). Unlike the other podocarps, *P. elongatus* produces epicormic sprouts that enable tolerance of fire and persistence in the fynbos biome that is prone to fire (Midgley *et al.* 1995), which makes it better adapted to the Mediterranean habitat. *Podocarpus elongatus* may be restricted to its Gondwanan origin and its disjunct distribution as a result of retreating to higher elevations to track favourable environmental conditions such as rising sea levels during the Last Glacial Maximum that lead to loss of coastal habitat (Geldenhuys 1992). *Podocarpus henkelii* is predicted to have a current distribution in summer rainfall regions with high rainfall in the eastern Limpopo province, the midlands of KwaZulu-Natal and the northern Eastern Cape province of South Africa.

The effects of climate change on the geographic distribution of a wide range of tree communities have been reported across the world (Bakkenes *et al.* 2002; Aitken *et al.* 2008; Essl *et al.* 2011; Aguilée *et al.* 2016; Burley *et al.* 2019; Kambach *et al.* 2019; Pavlović *et al.* 2019; Sedmáková *et al.* 2019; Fricke *et al.* 2022). *Podocarpus henkelii* was predicted to occupy the third largest geographic range under future climate emission scenarios (Fig. 2). Under future scenarios, *P. henkelii* may experience a contraction of its geographic distribution under relatively ‘mild’ climate change scenarios (e.g. RCP 4.5) yet will potentially expand its geographic distribution as temperatures rise (e.g. under RCP 8.5, Fig. 2). Its geographic range was predicted to contract in higher elevations and expand to lower elevations for both climate change emission scenarios. The southern expansion of its geographic area under both emission scenarios may suggest that the southern parts of South Africa will become hotter under climate change (Collier *et al.* 2008). Therefore, it is plausible that *P. henkelii* is a thermophilic species and can expand its distribution with increasing temperatures under climate change. This is also evident through the reduced geographic expansion of *P. henkelii* under RCP 8.5 in comparison to the cooler RCP 4.5 (Fig. 3). Twala *et al.* (2022) reported that *P. henkelii* was more heat tolerant than *A. falcatus.* Therefore, the ability of *P. henkelii* seeds to disperse across the grass matrix and/or establish in newly colonized areas could limit species expansion. In contrast, under both emission scenarios, *A. falcatus* and *P. latifolius* were predicted to expand to higher altitudes and lower longitudes to track favourable climatic conditions. The dispersal of *A. falcatus* and *P. latifolius* seeds by birds, bats, baboons and monkeys (Geldenhuys 1993; Negash 2003) will likely enable them to disperse to climatically favourable areas faster relative to *P. elongatus* and *P. henkelii.* The past responses of *P. elongatus* and *P. henkelii* to climate change indicate a tight synchronization with climate fluctuations, particularly in response to water availability. For *P. latifolius*, recruitment is less episodic than the other species thus making it less vulnerable to climatic instability. *Podocarpus elongatus* was predicted to lose suitable habitat in its southern distribution under both RCPs and expand northwards, particularly under RCP 4.5. However, shifting to higher elevations and longitudinal shifts seem to be the most common responses to climate change (Pavlović *et al.* 2019; Sedmáková *et al.* 2019).

Numerous studies have reported that plant species that occupy a large geographic range also occupy different environmental niches (Cardillo *et al.* 2019; Kambach *et al.* 2019; Quiroga and Souto 2022). We found that niche conservatism was evident between current and future RCPs for *A. falcatus, P. latifolius* and *P. henkelii* (Table 3). The current and future environmental niches under RCP 4.5 for *P. elongatus* also showed niche similarity; however, the RCP 4.5 → current, RCP 8.5 → Current and Current → RCP 8.5 comparisons showed niche divergence. The tendencies towards divergent environmental niches in podocarps cannot be explained by differences in the climate suitability alone. However, this divergence could be explained by differences in seed dispersal, establishment requirements, habitat use, competition and ecophysiological limits. There was no consistent trend in niche conservatism or divergence among the comparisons of current environmental niches and predicted future niches across the podocarp species examined. For example, *P. henkelii* may shift to new geographic areas under climate change/predicted emission scenarios, per niche conservatism, given the high niche overlap value and unfilling value (i.e. the future environmental niche space is available but currently unoccupied). Notably, there may be elevated propagule pressures that ultimately limit the dispersal of *P. henkelii* to sites with suitable environmental conditions, which will result in a reduced realized niche. It is also possible that *P. henkelii* may continue to spread in its geographic range even though the breadth of its environmental niche is predicted to decrease. Relative to the other three podocarps examined, *P. henkelii* had the lowest niche expansion value, which suggests that it will not expand into novel environmental niches when moving southwards geographically under climate change. On the other hand, the current environmental niche of *P. elongatus* diverged slightly from its future environment niche due to low niche overlap as a result of high niche expansion and unfilling (Table 4). This divergence may be attributed to differences in microhabitats and competition. In addition, the current and future environmental niches of *P. latifolius* showed low niche overlap, which was associated with low niche unfilling values. *Podocarpus latifolius* can occur within areas with variable gradients of soil moisture, temperature, rainfall and altitude along different latitudes, and it produces small seeds annually which are dispersed by birds (Geldenhuys 1993; Adie and Lawes 2009b). Moreover, frequent recruitment occurs due to lower seed susceptibility to desiccation and shade-tolerant seeds, which facilitates germination and reduces susceptibility to climate change. These characteristics indicate that *P. latifolius* is a vagile species. Despite this, results showed low niche expansion. This may be because *P. latifolius* currently occupies most of the suitable environmental niches available already, and there is no additional niche to expand into.

The ability of a species to occupy different environmental niches may have important implications for the understanding of the vulnerability of the species under climate change (Wiens and Graham, 2005). Previous studies recognized niche shifts in a variety of plant taxa such as *Picea sitchensis* (Aguilée *et al.* 2016), *Agave* spp. (Gómez-Ruiz and Lacher 2019) and *Betula utilis* (Hamid *et al.* 2019) under climate change, but the causes of these shifts are not well known. However, several studies have shown that some species will not be able to track favourable climate fast enough to escape climate change (Zhu *et al.* 2012; Cang *et al.* 2016; Burley *et al.* 2019). The combination of some niche expansion and unfilling for *A. falcatus* under both RCPs resulted in moderate niche stability under global change. The inability of *A. falcatus* to occupy environmental niches different from those of its current environmental niche may be due to a combination of post-dispersal mortality and loss in seed viability after harvesting, coupled with infrequent fruit production (Geldenhuys 1993; Negash 2003). Consequently, dispersal may be the main factor that governs whether *A. falcatus* reaches suitable habitats under global change.

Overall, we found that podocarp distribution is determined primarily by seasonal drought, likely due to the recalcitrant seeds of podocarps which are produced generally when rainfall is limited. The difference in the distribution of these podocarps underscores the variation of environmental conditions that they are adapted to and may also be a reflection on the differences in their physiology. This is particularly important as the physiological traits of podocarps may limit their distribution across environmental gradients (Brodribb and Hill, 1998, 1999; Brodribb 2011; Twala *et al.* 2022). Physiologically oriented distribution models should be useful to predict whether the physiological thresholds of podocarps are the mechanism responsible for podocarp distribution and not their reproductive means. Insights from this study are useful for ongoing conservation actions for South African podocarps by directing the re-introduction of these species in suitable habitats and guiding the establishment of effective networks of protected areas for future dispersal events.

## Data Availability

The datasets generated during and/or analysed during the current study are available in the Open Society Foundation repository, DOI 10.17605/OSF.IO/RNG5K (https://osf.io/rng5k/).

## Notes

**Plants, Ecosystems and Climate. Chief Editor:** Mary Heskel

## References

[CIT0001] Adie H , Lawes MJ. 2009a. Role reversal in the stand dynamics of an angiosperm–conifer forest: colonising angiosperms precede a shade-tolerant conifer in Afrotemperate forest. Forest Ecology and Management 258:159–168.

[CIT0002] Adie H , Lawes MJ. 2009b. Explaining conifer dominance in Afrotemperate forests: shade tolerance favours *Podocarpus latifolius* over angiosperm species. Forest Ecology and Management 259:176–186.

[CIT0003] Adie H , Lawes MJ. 2011. Podocarps in Africa: temperate zone relicts or rainforest survivors? In: Turner BL, Cernusak LA, Ecology of the Podocarpaceae in tropical forests. Washington, DC: Smithsonian Contributions to Botany. 79–100.

[CIT0004] Adie H , Kotze DJ, Lawes MJ. 2017. Small fire refugia in the grassy matrix and the persistence of Afrotemperate forest in the Drakensberg mountains. Scientific Reports 7:1–10. 28747738 10.1038/s41598-017-06747-2 PMC5529369

[CIT0005] Aguilée R , Raoul G, Rousset F, Ronce O. 2016. Pollen dispersal slows geographical range shift and accelerates ecological niche shift under climate change. Proceedings of the National Academy of Sciences 113:E5741–E5748. 10.1073/pnas.1607612113 PMC5047147 27621443

[CIT0006] Aitken SN , Yeama S, Holliday JA, Wang T, Curtis-McLane S. 2008. Adaptation, migration or extirpation: climate change outcomes for tree populations. Evolutionary Applications 1:95–111. 25567494 10.1111/j.1752-4571.2007.00013.x PMC3352395

[CIT0007] Allouche O , Tsoar A, Kadmon R. 2006. Assessing the accuracy of species distribution models: prevalence, kappa and the true skill statistic (TSS). Journal of Applied Ecology 43:1223–1232.

[CIT0008] Araújo MB , Ferri-Yáñez F, Bozinovic F, Marquet PA, Valladares F, Chown SL. 2013. Heat freezes niche evolution. Ecology Letters 16:1206–1219. 23869696 10.1111/ele.12155

[CIT0009] Bakkenes M , Alkemade JRM, Ihle F, Leemans R, Latour JB. 2002. Assessing effects of forecasted climate change on the diversity and distribution of European higher plants for 2050. Global Change Biology 8:390–407.

[CIT0010] Bernardi AP , Lauterjung MB, Mantovani A, dos Reis MS. 2020. Phylogeography and species distribution modeling reveal a historic disjunction for the conifer *Podocarpus lambertii*. Tree Genetics & Genomes 16:40.

[CIT0011] Biffin E , Brodribb TJ, Hill RS, Thomas P, Lowe AJ. 2012. Leaf evolution in Southern Hemisphere conifers tracks the angiosperm ­ecological radiation. Proceedings of the Royal Society B 279:341–348. 21653584 10.1098/rspb.2011.0559 PMC3223667

[CIT0012] Boria RA , Olson LE, Goodman SM, Anderson RP. 2014. Spatial filtering to reduce sampling bias can improve the performance of ecological niche models. Ecological Modelling 275:73–77.

[CIT0013] Bond WJ. 1989. The tortoise and the hare: ecology of angiosperm dominance and gymnosperm persistence. Biological Journal of the Linnean Society 36:227–249.

[CIT0014] Brodribb TJ. 2011. A functional analysis of podocarp ecology. In: Turner BL, Cernusak LA, eds. Ecology of the Podocarpaceae in tropical forests, Smithsonian Contributions to Botany, No. 95. Washington, DC: Smithsonian Institution Scholarly Press, 165–173.

[CIT0015] Brodribb TJ , Hill RS. 1998. The photosynthetic drought physiology of a diverse group of southern hemisphere conifer species is correlated with minimum seasonal rainfall. Functional Ecology 12:465–471.

[CIT0016] Brodribb TJ , Hill RS. 1999. The importance of xylem constraints in the distribution of conifer species. New Phytologist 143:365–372.

[CIT0017] Brodribb T , Hill RS. 2000. Increases in water potential gradient reduce xylem conductivity in whole plants: evidence from a low-pressure conductivity method. Plant Physiology 123:1021–1028. 10889251 10.1104/pp.123.3.1021 PMC59065

[CIT0018] Brodribb T , Hill RS. 2004. The rise and fall of the Podocarpaceae in Australia—a physiological explanation. In: The evolution of plant physiology. Amsterdam and Boston: Elsevier Academic Press, 381–399.

[CIT0019] Brodribb TJ , Feild TS, Jordan GJ. 2007. Leaf maximum photosynthetic rate and venation are linked by hydraulics. Plant Physiology 144:1890–1898. 17556506 10.1104/pp.107.101352 PMC1949879

[CIT0020] Broennimann O , Treier UA, Müller-Schärer H, Thuiller W, Peterson AT, Guisan A. 2007. Evidence of climatic niche shift during biological invasion. Ecology Letters 10:701–709. 17594425 10.1111/j.1461-0248.2007.01060.x

[CIT0021] Broennimann O , Fitzpatrick MC, Pearman PB, Petitpierre B, Pellissier L, Yoccoz NG, Thuiller W, Fortin M-F, Randin C, Zimmermann NE, et al. 2012. Measuring ecological niche overlap from occurrence and spatial environmental data. Global Ecology and Biogeography 21:481–497.

[CIT0022] Broennimann O , Di Cola V, Guisan A. 2020. ecospat: Spatial Ecology Miscellaneous Methods. R Packag version 31. https://CRANR-project.org/package=ecospat.

[CIT0023] Burley H , Beaumont LJ, Ossola A, Baumgartner JB, Gallagher R, Laffan S, Esperon Rodriguez M, Manea A, Leishman MR. 2019. Substantial declines in urban tree habitat predicted under climate change. Science of the Total Environment 685:451–462. 31176230 10.1016/j.scitotenv.2019.05.287

[CIT0024] Cang FA , Wilson AA, Wiens JJ. 2016. Climate change is projected to outpace rates of niche change in grasses. Biology Letters 12:20160368. 27677813 10.1098/rsbl.2016.0368 PMC5046922

[CIT0025] Cardillo M , Dinnage R, McAlister W. 2019. The relationship between environmental niche breadth and geographic range size across plant species. Journal of Biogeography 46:97–109.

[CIT0026] Chiariello N. 1984. Leaf energy balance in the wet lowland tropics. In: Medina E, Mooney HA, Vázquez-Yánes C, eds. Physiological ecology of plants of the wet tropics. Proc. Int. Symp. Tasks for Vegetation Science 12. The Hague: Dr. W. Junk Publishers, 85–98.

[CIT0027] Christenhusz MJ , Byng JW. 2016. The number of known plants species in the world and its annual increase. Phytotaxa 261:201–217.

[CIT0028] Collier P , Conwa G, Venables T. 2008. Climate change and Africa. Oxford Review of Economic Policy 24:337–353.

[CIT0029] Colyn RB , Ehlers Smith DA, Ehlers Smith YC, Smit-Robinson H, Downs CT. 2020. Predicted distributions of avian specialists: a framework for conservation of endangered forests under future climates. Diversity and Distributions 26:652–667.

[CIT0030] Contreras DL , Duijnstee IAP, Ranks S, Marshall CR, Looy CV. 2017. Evolution of dispersal strategies in conifers: functional divergence and convergence in the morphology of diaspores. Perspective in Plant Ecology, Evolution and Systematics 24:93–117.

[CIT0031] Coomes DA , Bellingham PJ. 2011. Temperate and tropical podocarps: how ecologically alike are they? In: Turner BL, Cernusak LA, eds. Ecology of the Podocarpaceae in tropical forests. Washington, DC: Smithsonian Contributions to Botany, 119–140.

[CIT0032] Di Cola V , Broennimann O, Petitpierre B, Breiner FT, d’Amen M, Randin C, Engler R, Pottier J, Pio D, Dubuis A, et al. 2017. ecospat: an R package to support spatial analyses and modeling of species niches and distributions. Ecography 40:774–787.

[CIT0033] Dodd MC , Staden JV, Smith MT. 1989a. Seed development in *Podocarpus henkelii*: an ultrastructural and biochemical study. Annals of Botany 64:297–310.

[CIT0034] Dodd MC , Staden JV, Smith MT. 1989b. Seed germination in *Podocarpus henkelii*: an ultrastructural and biochemical study. Annals of Botany 64:569–579.

[CIT0035] Eeley HA , Lawes MJ, Piper SE. 1999. The influence of climate change on the distribution of indigenous forest in KwaZulu-Natal, South Africa. Journal of Biogeography 26:595–617.

[CIT0036] Elith J , Leathwick JR. 2009. Species distribution models: ecological explanation and prediction across space and time. Annual Review of Ecology, Evolution, and Systematics 40:677–697.

[CIT0037] Elith J , Graham CH, Anderson RP, Dudı´k M, Ferrier S, Guisan A, Hijmans RJ, Huettmann F, Leathwick JR, Lehmann A, et al. 2006. Novel methods improve prediction of species’ distributions from occurrence data. Ecography 29:129–151.

[CIT0038] Elith J , Kearney M, Phillips S. 2010. The art of modelling range-shifting species. Methods in Ecology and Evolution 1:330–342.

[CIT0039] Essl F , Dullinger S, Plutzar C, Willner W, Rabitsch W. 2011. Imprints of glacial history and current environment on correlations between endemic plant and invertebrate species richness. Journal of Biogeography 38:604–614.

[CIT0040] Farjon A. 2001. World checklist and bibliography of conifers. Kew, Richmond, Surrey: Royal Botanic Gardens.

[CIT0041] Farjon A. 2010. A handbook of the world’s conifers, Vol 2. The Netherlands: Brill Leiden-Boston.

[CIT0042] Farjon A. 2018. The Kew review: conifers of the world. Kew Bulletin 73:8.

[CIT0043] Finch JM , Hill TR. 2008. A late quaternary pollen sequence from Mfabeni Peatland, South Africa: RECONSTRUCTING forest history in Maputaland. Quaternary Research 70:442–450.

[CIT0044] Fiorucci AS , Fankhauser C. 2017. Plant strategies for enhancing access to sunlight. Current Biology 27:R931–R940. 28898666 10.1016/j.cub.2017.05.085

[CIT0045] Fricke EC , Ordonez A, Rogers HS, Svenning JC. 2022. The effects of defaunation on plants’ capacity to track climate change. Science 375:210–214. 35025640 10.1126/science.abk3510

[CIT0046] Geldenhuys CJ. 1992. Disjunctions and distribution limits of forest species in the Southern Cape. South African Forestry Journal 161:1–13.

[CIT0047] Geldenhuys CJ. 1993. Reproductive biology and population structures of *Podocarpus falcatus* and *P. latifolius* in southern Cape forests. Botanical Journal of the Linnean Society 112:59–74.

[CIT0048] Geldenhuys CJ. 1994. Bergwind fires and the location pattern of forest patches in the southern cape landscape, South Africa. Journal of Biogeography 21:49–62.

[CIT0049] Givnish TJ. 1987. Comparative studies of leaf form: assessing the relative roles of selective pressures and phylogenetic constraints. New Phytologist 106:131–160.

[CIT0050] Glennon KL , Ritchie ME, Segraves KA. 2014. Evidence for shared broad-scale climatic niches of diploid and polyploid plants. Ecology Letters 17:574–582. 24818236 10.1111/ele.12259

[CIT0051] Gómez-Ruiz EP , Lacher Jr TE. 2019. Climate change, range shifts, and the disruption of a pollinator plant complex. Scientific Reports 9:1–10. 31575888 10.1038/s41598-019-50059-6 PMC6773846

[CIT0052] Grenouillet G , Buisson L, Casajus N, Lek S. 2011. Ensemble modelling of species distribution: the effects of geographical and environmental ranges. Ecography 34:9–17.

[CIT0053] Guisan A , Thuiller W. 2005. Predicting species distribution: offering more than simple habitat models. Ecology Letters 8:993–1009. 34517687 10.1111/j.1461-0248.2005.00792.x

[CIT0054] Guisan A , Tingley R, Baumgartner JB, Naujokaitis-Lewis I, Sutcliffe PR, Tulloch AI, Regan TJ, Brotons L, McDondald-Madden E, Mantyka-Pringle C, et al. 2013. Predicting species distributions for conservation decisions. Ecology Letters 16:1424–1435. 24134332 10.1111/ele.12189 PMC4280402

[CIT0055] Guisan A , Petitpierre B, Broennimann O, Daehler C, Kueffer C. 2014. Unifying niche shift studies: insights from biological invasions. Trends in Ecology & Evolution 29:260–269. 24656621 10.1016/j.tree.2014.02.009

[CIT0056] Guo C , Lek S, Ye S, Li W, Liu J, Li Z. 2015. Uncertainty in ensemble modelling of large-scale species distribution: effects from species characteristics and model techniques. Ecological Modelling 306:67–75.

[CIT0057] Hamid M , Khuroo AA, Charles B, Ahmad R, Singh CP, Aravind NA. 2019. Impact of climate change on the distribution range and niche dynamics of Himalayan birch, a typical treeline species in Himalayas. Biodiversity and Conservation 28:2345–2370.

[CIT0058] Hanley JA , McNeil BJ. 1982. The meaning and use of the area under a receiver operating characteristic (ROC) curve. Radiology 143:29–36. 7063747 10.1148/radiology.143.1.7063747

[CIT0059] He C , Zhao Y, Zhang J, Gao J. 2020. Chitosan oligosaccharide addition to Buddhist pine (*Podocarpus macrophyllus* (Thunb) Sweet) under drought: responses in ecophysiology and δ13C abundance. Forests 11:526.

[CIT0060] Hill RS , Brodribb T. 1999. Southern conifers in time and space. Australian Journal of Botany 47:639–696.

[CIT0061] Innes KP , Kelly D. 1992. Water potentials in native woody vegetation during and after a drought in Canterbury. New Zealand Journal of Botany 30:81–94.

[CIT0062] Jentsch H , Bobrowski M, Weidinger W. 2021. ClimDatDownloadR: downloads climate data from Chelsa and WorldClim. R package version 0.1.5.

[CIT0063] Kambach S , Lenoir J, Decocq G, Welk E, Seidler G, Dullinger S, Gégout J-C, Guisan A, Pauli H, Svenning J-C, et al. 2019. Of niches and distributions: range size increases with niche breadth both globally and regionally but regional estimates poorly relate to global estimates. Ecography 42:467–477.

[CIT0064] Karger DN , Conrad O, Böhner J, Kawohl T, Kreft H, Soria-Auza RW, Zimmermann NE, Linder HP, Kessler M. 2017. Climatologies at high resolution for the earth’s land surface areas. Scientific Data 4:1–20. 10.1038/sdata.2017.122 PMC5584396 28872642

[CIT0065] Kellman M , Meave J. 1997. Fire in the tropical gallery forests of Belize. Journal of Biogeography 24:23–34.

[CIT0066] Kitayama K , Aiba SI, Ushio M, Seino T, Fujiki Y. 2011. The ecology of podocarps in tropical montane forests of Borneo: distribution, population dynamics, and soil nutrient acquisition. In: Turner BL, Cernusak LA, Ecology of the Podocarpaceae in tropical forests. Smithsonian Contributions to Botany. 101–107.

[CIT0067] Klaus KV , Matzke NJ. 2020. Statistical comparison of trait-dependent biogeographical models indicates that Podocarpaceae dispersal is influenced by both seed cone traits and geographical distance. Systematic Biology 69:61–75. 31099388 10.1093/sysbio/syz034

[CIT0068] Krassilov VA. 1974. *Podocarpus* from the upper cretaceous of eastern Asia and its bearing on the theory of conifer evolution. Palaeontology 17:365–370.

[CIT0069] Leslie AB , Beaulieu JM, Rai HS, Crane PR, Donoghue MJ, Mathews S. 2012. Hemisphere scale differences in conifer evolutionary dynamics. Proceedings of the National Academy of Sciences of the USA 109:16217–16221. 22988083 10.1073/pnas.1213621109 PMC3479534

[CIT0070] Lusk CH , Grierson ER, Laughlin DC. 2019. Large leaves in warm, moist environments confer an advantage in seedling light interception efficiency. New Phytologist 223:1319–1327. 30985943 10.1111/nph.15849

[CIT0071] Martin GM , Bellouin N, Collins WJ, Culverwell ID, Halloran PR, Hardiman SC, Hinton TJ, Jones CD, McDonald RE, McLaren AJ, et al. 2011. The HadGEM2 family of Met Office Unified Model climate configurations. Geoscientific Model Development 4:723–757.

[CIT0072] Meinshausen M , Smith SJ, Calvin K, Daniel JS, Kainuma ML, Lamarque JF, Matsumoto S, Montzka SA, Raper SCB, Riahi K, et al. 2011. The RCP greenhouse gas concentrations and their extensions from 1765 to 2300. Climatic Change 109:213–241.

[CIT0073] Midgley JJ , Bond WJ, Geldenhuys CJ. 1995. The ecology of the Southern African conifers. In: Enright NJ, Hill RS, eds. Ecology of the Southern Conifers. Carltona: Melbourne University Press, 64–80.

[CIT0074] Migliore J , Lézine AM, Veuille M, Achoundong G, Tchiengué B, Boom AF, Monthe FK, Bouka GUD, Omondi SF, Wagura L, et al. 2022. Origin, persistence, and vulnerability to climate changes of *Podocarpus* populations in Central African Mountains. Forests 13:208.

[CIT0075] Mill RR. 1999. Towards a biogeography of the Podocarpaceae. In: IV International Conifer Conference 615, 137–147.

[CIT0076] Molloy BPJ. 1995. Manoao (Podocarpaceae), a new monotypic conifer genus endemic to New Zealand. New Zealand Journal of Botany 33:183–201.

[CIT0077] Moran EV. 2020. Simulating the effects of local adaptation and life history on the ability of plants to track climate shifts. AoB Plants 12:plaa008. 32128105 10.1093/aobpla/plaa008 PMC7046178

[CIT0078] Morley RJ. 2000. Origin and evolution of tropical rain forests. London: John Wiley and Sons.

[CIT0079] Morley RJ. 2011. Dispersal and paleoecology of tropical podocarps. In: Turner BL, Cernusak LA, eds. Ecology of the Podocarpaceae in tropical forests. Washington, DC: Smithsonian Contributions to Botany, 21–41.

[CIT0080] Negash L. 1995. *Indigenous trees of Ethiopia. Biology, uses and propagation techniques*. *Repro*. Umeå: SLU.

[CIT0081] Negash L. 2003. In situ fertility decline and provenance differences in the East African yellow wood (*Podocarpus falcatus*) measured through in vitro seed germination. Forest Ecology and Management 174:127–138.

[CIT0082] Neumann FH , Stager JC, Scott L, Venter HJ, Weyhenmeyer C. 2008. Holocene vegetation and climate records from lake Sibaya, KwaZulu-Natal (South Africa). Review of Palaeobotany and Palynology 152:113–128.

[CIT0083] Noel ARA , Van Staden J. 1976. Seed coat structure and germination in *Podocarpus henkelii*. Zeitschrift für Pflanzenphysiologie 77:174–186.

[CIT0084] Pang SE , De Alban JDT, Webb EL. 2021. Effects of climate change and land cover on the distributions of a critical tree family in the Philippines. Scientific Reports 11:1–13. 33432023 10.1038/s41598-020-79491-9 PMC7801684

[CIT0085] Pavlović L , Stojanović D, Mladenović E, Lakićević M, Orlović S. 2019. Potential elevation shift of the European beech stands (*Fagus sylvatica* L.) in Serbia. Frontiers in Plant Science 10:849. 31338099 10.3389/fpls.2019.00849 PMC6629902

[CIT0086] Peterson AT. 2011. Ecological niche conservatism: a time-structured review of evidence. Journal of Biogeography 38:817–827.

[CIT0087] Peterson AT , Soberón J, Sánchez-Cordero V. 1999. Conservatism of ecological niches in evolutionary time. Science 285:1265–1267. 10455053 10.1126/science.285.5431.1265

[CIT0088] Petitpierre B , Kueffer C, Broennimann O, Randin C, Daehler C, Guisan A. 2012. Climatic niche shifts are rare among terrestrial plant invaders. Science 335:1344–1348. 22422981 10.1126/science.1215933

[CIT0089] Quiroga MP , Premoli AC. 2007. Genetic patterns in *Podocarpus parlatorei* reveal the long-term persistence of cold-tolerant elements in the southern Yungas. Journal of Biogeography 34:447–455.

[CIT0090] Quiroga MP , Souto CP. 2022. Ecological niche modeling, niche overlap, and good old Rabinowitz’s rarities applied to the conservation of gymnosperms in a global biodiversity hotspot. Landscape Ecology 37:2571–2588.

[CIT0091] Quiroga MP , Mathiasen P, Iglesias A, Mill RR, Premoli AC. 2016. Molecular and fossil evidence disentangle the biogeographical history of Podocarpus, a key genus in plant geography. Journal of Biogeography 43:372–383.

[CIT0092] Quiroga MP , Premoli AC, Kitzberger T. 2018. Niche squeeze induced by climate change of the cold tolerant subtropical montane *Podocarpus parlatorei*. Royal Society Open Science 5:180513. 30564385 10.1098/rsos.180513 PMC6281919

[CIT0093] Rödder D , Engler JO. 2011. Quantitative metrics of overlaps in Grinnellian niches: advances and possible drawbacks. Global Ecology and Biogeography 20:915–927.

[CIT0094] Schoener TW. 1968. The Anolis lizards of Bimini: resource partitioning in a complex fauna. Ecology 49:704–726.

[CIT0095] Sedmáková D , Sedmák R, Bosela M, Ježík M, Blaženec M, Hlásny T, Marušák R. 2019. Growth climate responses indicate shifts in the competitive ability of European beech and Norway spruce under recent climate warming in East-Central Europe. Dendrochronologia 54:37–48.

[CIT0096] Soberón J. 2007. Grinnellian and Eltonian niches and geographic distributions of species. Ecology Letters 10:1115–1123. 17850335 10.1111/j.1461-0248.2007.01107.x

[CIT0097] Synes NW , Osborne PE. 2011. Choice of predictor variables as a source of uncertainty in continental-scale species distribution modelling under climate change. Global Ecology and Biogeography 20:904–914.

[CIT0098] Tesfamariam BG , Gessesse B, Melgani F. 2022. MaxEnt-based modeling of suitable habitat for rehabilitation of Podocarpus forest at landscape-scale. Environmental Systems Research 11:1–12.

[CIT0099] Thuiller W , Georges D, Gueguen M, Engler R, Breiner F. 2021. Biomod2: Ensemble platform for species distribution modeling. R package version 3.5.1. https://cran.r-project.org/package=biomod2.

[CIT0100] Twala TC , Witkoski ETF, Fisher JT. 2022. The effects of heat and drought stress on the ecophysiological responses and growth of *Afrocarpus falcatus* and *Podocarpus henkelii* seedlings. South African Journal of Botany 149:268.

[CIT0101] Van Vuuren DP , Stehfest E, den Elzen MG, Kram T, van Vliet J, Deetman S, Isaac M, Klein Goldewijk K, Hof A, Mendoza Beltran A, et al. 2011. RCP2. 6: exploring the possibility to keep global mean temperature increase below 2˚C. Climatic Change 109:95–116.

[CIT0102] Warren DL , Glor RE, Turelli M. 2008. Environmental niche equivalency versus conservatism: quantitative approaches to niche evolution. Evolution; International Journal of Organic Evolution 62:2868–2883. 18752605 10.1111/j.1558-5646.2008.00482.x

[CIT0103] Warren DL , Glor RE, Turelli M. 2010. ENMTools: a toolbox for comparative studies of environmental niche models. Ecography 33:607–611.

[CIT0104] Warren DL , Beaumont LJ, Dinnage R, Baumgartner J. 2019. New methods for measuring ENM breadth and overlap in environmental space. Ecography 42:444–446.

[CIT0105] White F. 1981. The history of the Afromontane archipelago and the scientific need for its conservation. African Journal of Ecology 19:33–54.

[CIT0106] Wiens JJ. 2004. Speciation and ecology revisited: phylogenetic niche conservatism and the origin of species. Evolution 58:193–197. 15058732 10.1111/j.0014-3820.2004.tb01586.x

[CIT0107] Wiens JJ , Graham CH. 2005. Niche conservatism: integrating evolution, ecology, and conservation biology. Annual Review of Ecology, Evolution, and Systematics 36:519–539.

[CIT0108] Wiens JA , Stralberg D, Jongsomjit D, Howell CA, Snyder MA. 2009. Niches, models, and climate change: assessing the assumptions and uncertainties. Proceedings of the National Academy of Sciences 106(supplement_2): 19729–19736. 10.1073/pnas.0901639106 PMC2780938 19822750

[CIT0109] Wiens JJ , Ackerly DD, Allen AP, Anacker BL, Buckley LB, Cornell HV, Damschen EI, Davies TJ, Grytnes J-A, Harrison SP, et al. 2010. Niche conservatism as an emerging principle in ecology and conservation biology. Ecology Letters 13:1310–1324. 20649638 10.1111/j.1461-0248.2010.01515.x

[CIT0110] Zhu K , Woodall CW, Clark JS. 2012. Failure to migrate: lack of tree range expansion in response to climate change. Global Change Biology 18:1042–1052.

[CIT0111] Zurell D , Zimmermann NE, Gross H, Baltensweiler A, Sattler T, Wüest RO. 2020. Testing species assemblage predictions from stacked and joint species distribution models. Journal of Biogeography 47:101–113.

